# Deep RNA sequencing of pectoralis muscle transcriptomes during late-term embryonic to neonatal development in indigenous Chinese duck breeds

**DOI:** 10.1371/journal.pone.0180403

**Published:** 2017-08-03

**Authors:** Chunhong Zhu, Weitao Song, Zhiyun Tao, Hongxiang Liu, Wenjuan Xu, Shuangjie Zhang, Huifang Li

**Affiliations:** Jiangsu Institute of Poultry Science, Yangzhou, Jiangsu Province, People’s Republic of China; INIA, SPAIN

## Abstract

Pectoral muscle (PM) comprises an important component of overall meat mass in ducks. However, PM has shown arrested or even reduced growth during late embryonic development, and the molecular mechanisms underlying PM growth during the late embryonic to neonatal period in ducks have not been addressed. In this study, we characterized potential candidate genes and signaling pathways related to PM development using RNA sequencing of PM samples selected at embryonic days (E) 21 and 27 and 5 days post-hatch (dph) in two duck breeds (Gaoyou and Jinding ducks). A total of 393 differentially expressed genes (DEGs) were identified, which showed higher or lower expression levels at E27 compared with E21 and 5 dph, reflecting the pattern of PM growth rates. Among these, 43 DEGs were common to all three time points in both duck breeds. These DEGs may thus be involved in regulating this developmental process. Specifically, KEGG pathway analysis of the 393 DEGs showed that genes involved with different metabolism pathways were highly expressed, while genes involved with cell cycle pathways showed lower expression levels at E27. These DEGs may thus be involved in the mechanisms responsible for the phenomenon of static or decreased breast muscle growth in duck breeds during the late embryonic period. These results increase the available genetic information for ducks and provide valuable resources for analyzing the mechanisms underlying the process of PM development.

## Introduction

The advent of modern agriculture in the early 20^th^ century led to intensive genetic selection for meat- and egg-production traits in domesticated ducks. Meat-type and egg-type duck breeds have undergone different genetic selection processes, and thus display significant genetic differences in terms of muscle growth rates and egg production, whilst maintaining consistent developmental processes, e.g. in muscle development pattern. Gaoyou and Jinding ducks comprise important indigenous Chinese meat-type and egg-type duck breeds (average bodyweight approximately 2.48 kg and 1.54 kg at 70 days old for Gaoyou and Jinding ducks, respectively) (Animal Genetic Resources in China-Poultry, 2011)[1]. The different genetic backgrounds in terms of muscle between Gaoyou and Jinding ducks provides a potential model for identifying the mechanisms involved in the muscle-development process. We previously compared body weight, leg and pectoral muscle (PM) masses, and myofiber characteristics between Gaoyou and Jinding ducks during early development[2–4]. We found that the increase in PM mass slowed from embryonic day (E) 21, with no increase from E21 to E25, followed by a slight decrease in mass before hatching (E27), and then an slow increase from hatching to day 7 post-hatching[3]. Jing et al.[5] and Chen et al.[6] also observed a similar developmental pattern of PM in a meat-type duck breed. However, this process differs from that observed in other animals, such as pig fetuses of the corresponding age, in which the mass of various muscles increased considerably. According to Chen et al.[6], the decrease in PM mass was caused by protein catabolism due to energy deficiency during late-term incubation and the mobilization of skeletal muscle protein to supply most of the endogenous glutamine. They also investigated and discussed the possible key genes involved in signaling pathways. We believe the above record was just part causality. Skeletal muscle development is a complex process involving myoblast proliferation, differentiation into multinucleated myotubes, and the eventual formation of mature muscle fibers during the incubation period in avian species[7]. All the genes and pathways involved in these processes could be differentially regulated in PM during the late embryonic to neonatal periods. In this study, we analyzed the respective PM transcriptomes in Gaoyou and Jinding ducks with different PM phenotypes and genetic backgrounds, to screen for genes and mechanisms involved in PM development in ducks before and after hatching.

High-throughput sequencing technologies are rapidly evolving, and their application to transcriptome analysis (RNA-Seq), together with adapted bioinformatic tools, allow unprecedented exploration of the transcriptome in terms of accuracy and data insights[8]. The duck (*Anas platyrhynchos*) genome has been sequenced[9] and the draft genome (BGI_duck_1.0) is now publicly available (http://www.ensembl.org/Anas_platyrhynchos/Info/Index), thus improving the accuracy of duck RNA-Seq analysis, and facilitating the identification and functional exploration of differentially expressed genes (DEGs) in ducks. Some previous RNA-Seq studies have analyzed growth traits in ducks[10,11]. The present study aimed to test our hypothesis using RNA-Seq to compare PM gene expression patterns at different time points during late-term embryonic to neonatal development in Gaoyou and Jinding ducks. We constructed eighteen polyA mRNA-seq libraries, including nine from Gaoyou duck PM at E21 and E27, and at 5 days post-hatching (dph) (three libraries at each developmental stage), and nine libraries from Jinding duck PM at the same time points (three libraries at each developmental stage). We performed an integrated analysis of the DEGs detected in this work to identify key genes and pathways affecting early PM development in ducks.

## Materials and methods

### Animals and PM sample collection

Two indigenous Chinese duck breeds, meat-type Gaoyou and egg-type Jinding ducks, were chosen for the present study because of their differential growth phenotypes. Two hundred half-sib fertile eggs of Gaoyou and Jinding ducks, respectively, from Jiangsu Gaoyou Duck Group (Jiangsu, China) were incubated in a standard commercial incubator. A total of 20–24 eggs were sampled from each breed on incubation days E21 and E27, and the muscles were collected from the left side. The anterior region of the left side was sampled and frozen immediately in liquid nitrogen and then stored at –80°C for RNA isolation. After direct examination of the gonad tissue and measuring the body weight and PM mass, six E21 female samples (3 Gaoyou, 3 Jinding) and six E27 female samples (3 Gaoyou, 3 Jinding) were randomly selected for subsequent analysis. Duck poults were reared from hatching to 5 days old in heated battery/brooder units. The temperature was maintained at 31°C at hatching, and was decreased gradually to 25°C until day 5. Light was provided continuously throughout the experiment. Ducklings were provided *ad libitum* with water and a pellet diet that met or exceeded NRC (National Research Council, 1994) nutrient recommendations (metabolizable energy 2.82 Mcal/kg, crude protein 22%) within 24 h after hatching. At 5 dph, 10 female ducklings selected randomly from each breed were re-fed for 1.5 h after a 16 h fast and then killed by anesthesia and exsanguination. Body weight and PM mass were measured, and six 5 dph female samples (3 Gaoyou, 3 Jinding) were selected for later analysis. All protocols involving the use of animals received prior approval from Jiangsu Institute of Poultry Science Animal Care and Use Committees.

### RNA isolation, library preparation, and sequencing

Total RNA was isolated from approximately 30 mg samples of PM tissue using Trizol^®^ reagent according to the manufacturer’s instructions (Invitrogen, Carlsbad, CA, USA). RNA degradation and contamination were assessed on 1% agarose gels. RNA concentration was measured using a Qubit^®^ RNA Assay Kit in a Qubit^®^ 2.0 Fluorometer (Life Technologies, CA, USA). RNA purity and integrity were checked using a Nano Photometer^®^ spectrophotometer (Implen, Westlake Village, CA, USA) and an RNA Nano 6000 Assay Kit (Bioanalyzer 2100 system; Agilent Technologies, Palo Alto, CA, USA), respectively.

A total amount of 3 μg RNA was used as input material for the RNA sample preparations. Finally, 18 samples with RNA integrity number (RIN) values >8.8 were used for library construction. Sequencing libraries were generated using an Illumina Tru Seq^™^ RNA sample preparation kit (Illumina, San Diego, CA, USA) following the manufacturer’s recommendations, and index codes were added to attribute a sequence to each sample. Briefly, polyA RNA mRNA was purified from total RNA using poly-T oligo-attached magnetic beads. Fragmentation was carried out using divalent cations at elevated temperature in NEBNext First Strand Synthesis Reaction Buffer (5×). First strand cDNA was synthesized using random hexamer primers and M-MuLV reverse transcriptase (RNase H−). Second strand cDNA synthesis was subsequently performed using DNA polymerase I and RNase H. Remaining overhangs were converted into blunt ends via exonuclease/polymerase activities. After adenylation of 3′ ends of DNA fragments, a NEB Next Adaptor with hairpin loop structure was ligated to prepare for hybridization. To select cDNA fragments of 150–200 bp in length, library fragments were purified with an AMPure XP system (Beckman Coulter, Beverly, MA, USA), followed by 3 μl USER Enzyme (NEB) with size-selected, adaptor-ligated cDNA at 37°C for 15 min followed by 5 min at 95°C before PCR. PCR was performed for 10 cycles with Phusion High-Fidelity DNA polymerase, Universal PCR primers and Index (X) Primer. PCR products were finally purified (AMPure XP system) and library quality was assessed using an Agilent Bioanalyzer 2100 system.

Clustering of the index-coded samples was performed on a cBot Cluster Generation System using TruSeq PE Cluster Kit v3-cBot-HS (Illumina) according to the manufacturer’s instructions. After cluster generation, the library preparations were sequenced using an Illumina HiSeq 2500 platform and 125-bp paired-end reads were generated.

### Quality control for paired-end reads

Raw data (raw reads) in FASTQ format were first processed using in-house perl scripts. The filtered data (filtered reads) were obtained by removing reads containing adapter or poly-N and low-quality reads from the raw data. At the same time, quality parameters for filtered data including Q20 (proportion of bases with a phred base-quality score >20; i.e., proportion of read bases with error rate <1%), Q30 (proportion of bases with a phred base-quality score >30; i.e., proportion of read bases with error rate <0.1%), GC content, and sequence-duplication level were used for data filtering (S1 Table). All the subsequent analyses were carried out using high-quality filtered data.

#### Sequence reads alignment to duck reference genome and annotated transcripts

Reference genome and gene model annotation files were downloaded from the duck genome website (ftp://ftp.ensembl.org/pub/release-80/fasta/anas_platyrhynchos/dna/). An index of the reference genome was built using Bowtie v2.0.6 and paired-end clean reads were aligned to the reference genome using TopHat v2.0.12 (http://tophat.cbcb.umd.edu/), set to mismatch = 2, with all other settings on the default parameters. TopHat was chosen as the mapping tool because it can generate a database of splice junctions based on the gene model annotation file, and thus give a better mapping result than other non-splice mapping tools. The detailed alignment information is presented in S1 Table, including total numbers of reads, mapped reads, and unique mapped reads.

### Quantification and differential expression analysis of transcripts

HTSeq v0.6.1 (http://www-huber.embl.de/users/anders/HTSeq) was used to count the read numbers mapped to each gene. RPKM (reads per kilobase of exon model per million mapped reads) were calculated based on the length of the gene and read counts mapped to this gene. This is currently the most commonly used method for estimating gene expression[12].

Differential expression was analyzed using the DESeq R package (1.10.1). DESeq provides statistical routines to determine differential expression in digital gene expression data using a model based on the negative binomial distribution. The resulting *P* values were adjusted using the Benjamini and Hochberg approach for controlling the false discovery rate[13]. Differential expression at the mRNA level was analyzed using Cuffdiff v2.2.1. A corrected *P* value of 0.05 was set as the threshold for significant differential expression.

### GO and gene functional analysis of DEGs

The Goseq R package implemented GO enrichment analysis of DEGs, in which gene length bias was corrected. GO terms with corrected *P*-values <0.05 were considered significantly enriched among the DEGs. We used KOBAS software to test the statistical enrichment of DEGs in KEGG pathways.

### qRT-PCR

To validate the repeatability and reproducibility of the gene expression data obtained by RNA sequencing in ducks, qRT-PCR was carried out on 16 selected DEGs from total RNA as used for RNA-Seq and superscript II reverse transcriptase (Invitrogen)-synthesized first-strand cDNA (primers shown in S2 Table). Gene-specific primers were designed by Primer 3 (http://fokker.wi.mit.edu/primer3/input.htm) and validated using Oligo 6.0. Primer sequences are shown in S2 Table. mRNA levels of the DEGs were normalized against the housekeeping genes glyceraldehyde 3-phosphate dehydrogenase (*GAPDH*) and beta-actin (*ACTB*) in the corresponding samples. qRT-PCR was carried out in triplicate with Super Real PreMix (SYBR Green) (FP204-01) master mix on a Real-Time PCR Detection System (Tiangen Biotech Co., Ltd., Beijing, China) and an ABI PRISM 7900 Sequence Detection System (Applied Biosystems) using the following program: 95°C for 10 min; 45 cycles of 95°C for 10 s, 60°C for 10 s, 72°C for 10 s, and 72°C for 6 min.

## Results

### Draft reads

To identify potential candidate genes affecting early PM development in ducks (especially at E27), we examined gene expression profiles in PM samples from Gaoyou and Jinding ducks during late-term embryonic to neonatal development using RNA-Seq. RNA was prepared from three PM samples from different developmental stages (E21 and E27 and 5 dph). Eighteen cDNA libraries were then constructed and subjected to Illumina deep sequencing for both duck breeds. There were about 45 million filtered reads, the total mapped rate was 58.58%–64.82%, and the uniquely mapped rate was 56.21%–63.55%. All Q20 values (see Methods) were >94.015%, and the Q30 value was up to 91.015%. A schematic of the Illumina deep sequencing and analysis procedures is shown in S1 Table.

### DEGs in PM among different developmental periods

**Fig 1:**
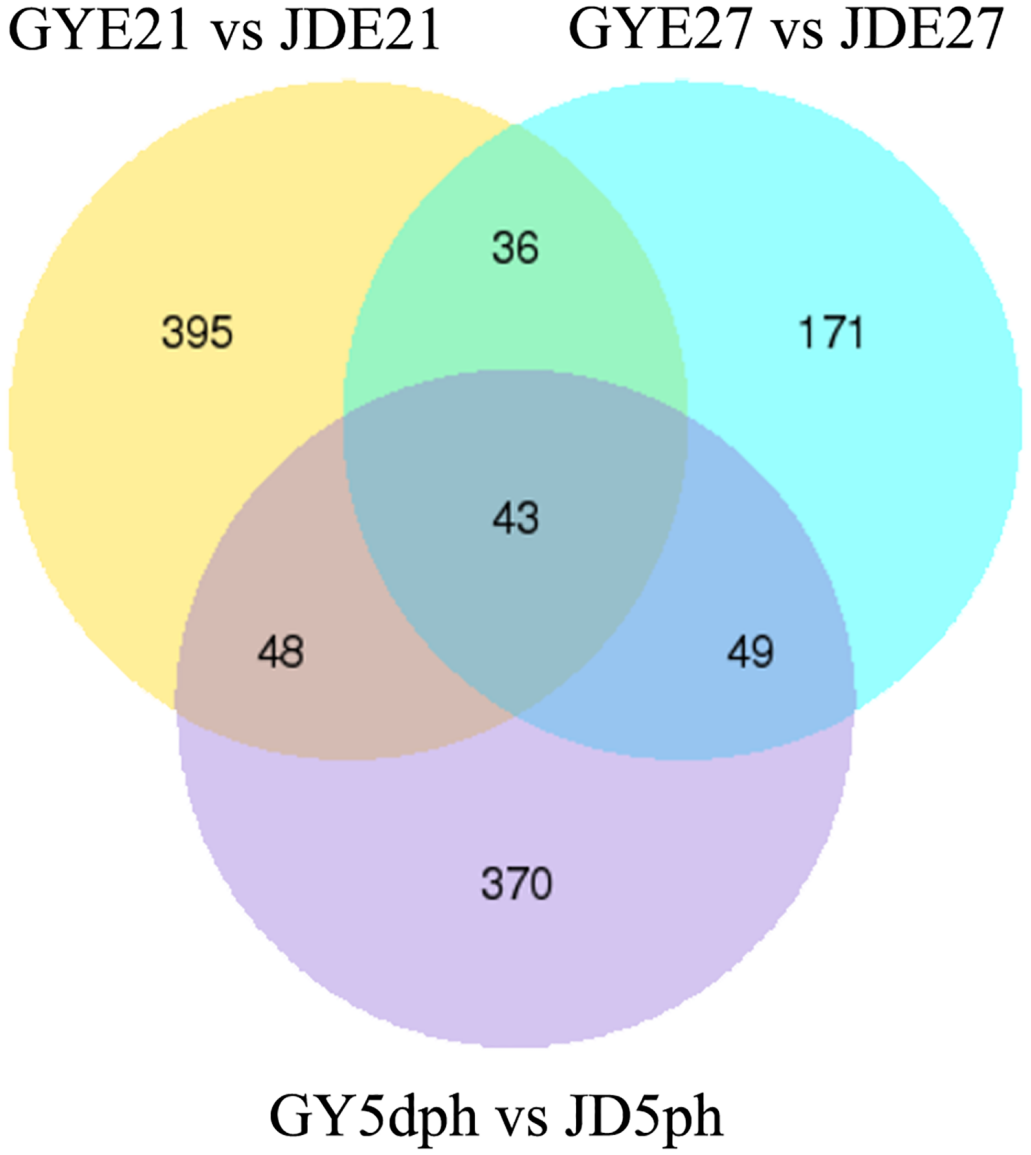
Venn diagram of differentially expressed genes (DEGs) among three developmental time points (E21 and E27 and 5 dph) and between two duck breeds. GY, Gaoyou duck; JD, Jinding duck.

**Fig 2:**
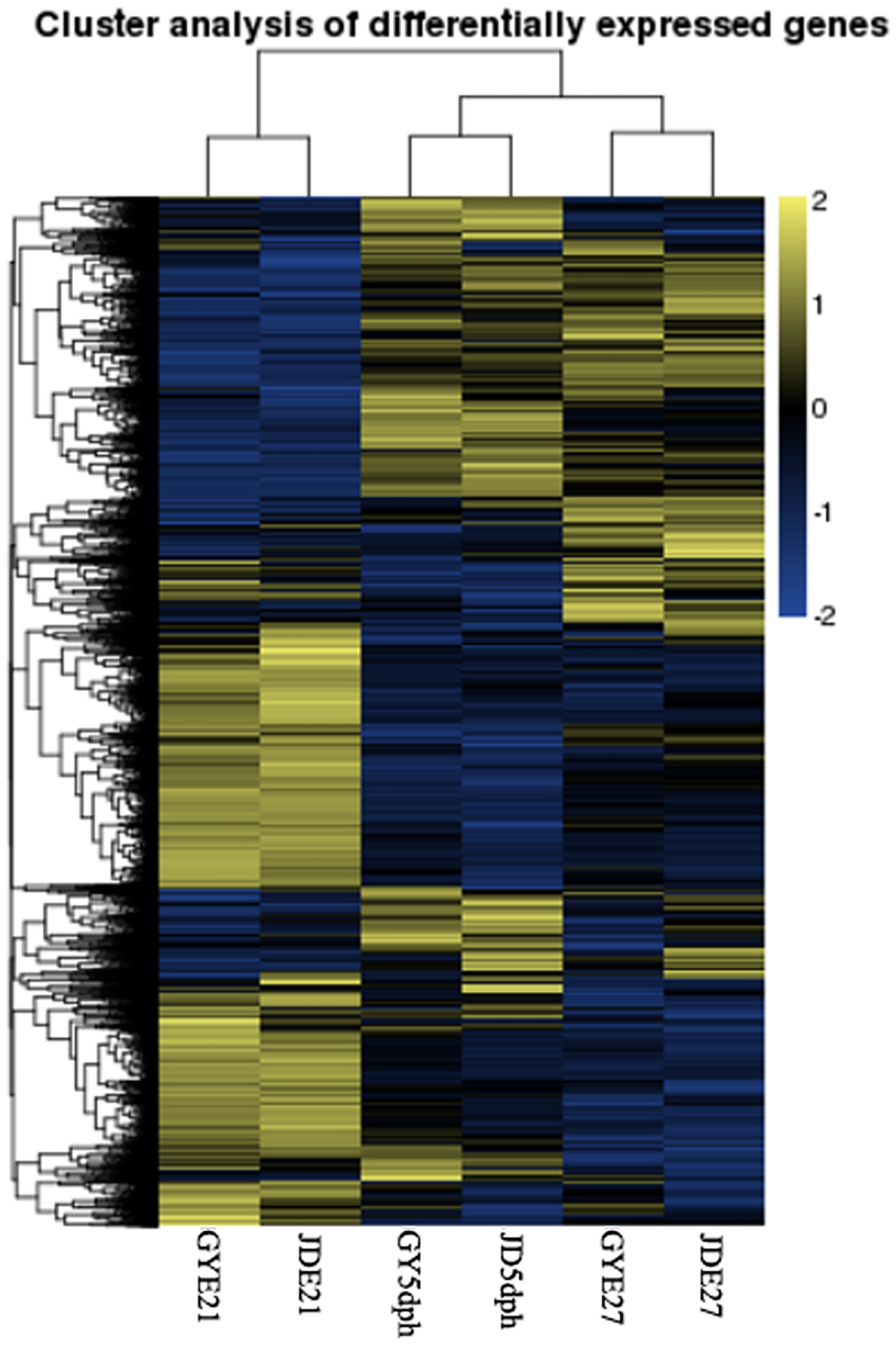
Cluster analysis of differentially expressed genes (DEGs). Expression profiles of DEGs at the same developmental time node in both duck breeds were combined. Yellow indicates high-expressed genes, and blue indicates low-expressed genes. GY, Gaoyou duck; JD, Jinding duck. Log_10_ (RPKM+1).

DEGs, expressed in RPKM, were compared among three developmental time points (E21 and E27 and 5 dph) and between the two duck breeds (Figs 1 and 2). The results of cluster analysis of DEGs are shown in Fig 2, with the same developmental time nodes superimposed. There were 522 DEGs between Gaoyou and Jinding ducks at E21, including 146 novel genes. There were 299 DEGs and 81 novel genes at E27, and 510 DEGs and 118 novel genes at 5 dph. A total of 43 DEGs were shared by both breeds at all time points, including19 novel genes (Fig 1 and S3 Table).

PM growth showed a static or decreasing trend at E27 compared with E21 and 5 dph[4–6]. In this study, we focused on DEGs that showed higher or lower expression levels at E27 compared with E21 and 5 dph in both duck breeds. Among 1002 DEGs in Gaoyou ducks, 528 were more highly expressed at E21 and 5 dph compared with E27 (termed ‘up-regulated’), and 474 genes were more highly expressed at E27 (termed ‘down-regulated’), while among 663 DEGs in Jinding ducks, 359 were up-regulated and 304 were down-regulated. Cuffdiff analysis identified 776 DEGs that were up-regulated at E21 and 5 dph compared with E27, and 138 DEGs that were down-regulated at the mRNA level. A total of 306 DEGs were highly expressed at E27, while 339 DEGs were up-regulated in Jinding ducks.

**Fig 3:**
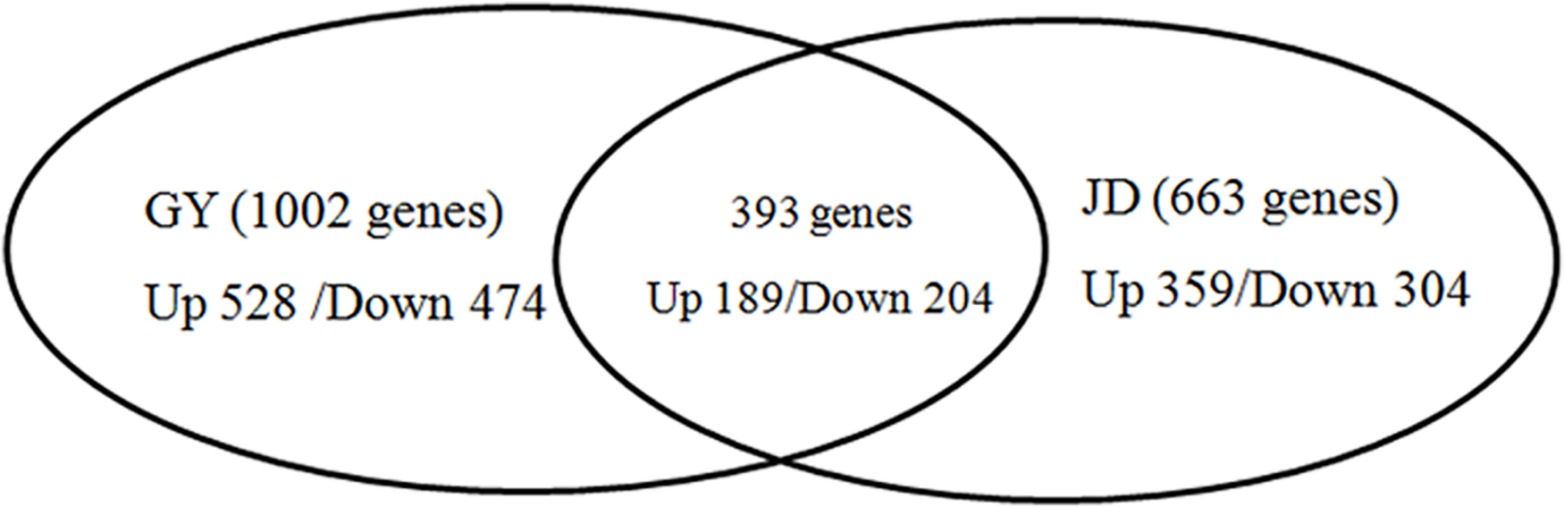
Numbers of genes that were differentially expressed in PM among E21 and E27 and 5 dph. GY, Gaoyou ducks; JD, Jinding ducks.

We further considered the 393 genes with expression patterns in line with the muscle development curve in both breeds (S4 Table). Among the 393 genes, 204 genes were more highly expressed at E27 compared with E21 and 5 dph, while 189 genes showed lower expression levels at E27 (Fig 3). Regarding the functions of these genes, they included several important genes significantly related to muscle development. Three genes, *TNNT2* (troponin T), *TNNI1* (troponin I) and *TNNC1* (troponin C) showed the lowest expression levels at E27. Expression levels of these three genes in Gaoyou ducks were decreased 261.65, 10.22, and 30.44 times compared with that at E21, with a slight increase at 5 dph (6.42, 1.75, 6.63 times compared with E27). The expression trends and fold changes of these genes in Jinding ducks were similar. This subset of DEGs also included genes with relevant functions (as discussed later) (S4 Table) such as *MUSTN1* (musculoskeletal embryonic nuclear protein 1), *MYBPH* (myosin-binding protein H), *ANXA5* (annexin A5), *MYOZ1* (myozenin-1), *FNDC5* (fibronectin type III domain-containing protein 5), *IGF1* (insulin-like growth factor [IGF]) I), *IGF2BP3* (IGF2 mRNA-binding protein 3), *CENPF* (centromere protein F), and *SYNEM* (synemin).

Cuffdiff analysis revealed 275 genes with expression levels in line with the PM development curves in both Gaoyou and Jinding ducks, including 196 up-regulated and 79 down-regulated DEGs. These genes showed partial overlap with the 393 DEGs detected by DESeq R package (159 DEGs), as well as 116 new genes (including 28 novel genes without gene descriptions). Regarding the functions of these 88 DEGs, two (*CENPE*, *FOXO3*) may be involved in muscle development.

### DEGs among different developmental periods

A total of 5422 genes were differentially expressed between E27 and E21 in Jinding ducks, and 5174 in Gaoyou ducks. A total of 4161 DEGs were shared by both breeds during this developmental period, comprising 76.74% and 80.42%, of the DEGs in each breed, respectively. There were 1813 DEGs compared between 5 dph and E27 in Jinding ducks, and 2308 in Gaoyou ducks, with 1130 shared DEGs, accounting for > 50% of the DEGs (S5 Table). These results indicated that high percentages of DEGs were shared by both duck breeds at these two time periods.

### Validation of gene expression level

The threshold that corresponded to reliable results was assayed by quantitative reverse transcription-polymerase chain reaction (qRT-PCR). Comparison of the RPKM values for 16 genes (selected from the total of 43 DEGs shared by all time points and both duck breeds and the above 393 DEGs) by *ΔΔ*Ct showed a strong relationship. The Pearson correlation values were >0.70 (Table 1). These results showed acceptable consistency between qPCR and fold change of DEGs in the transcriptome analysis.

**Table 1:** Correlation between RPKM and *ΔΔ*Ct of gene expression profiles by qRT-PCR.

Gene symbol	*P*-correlation	Gene id	Description
*F16P1*^#^	0.81	sp|P00636|	Fructose-1,6-bisphosphatase 1
*COL1A1*^#^	0.79	sp|P02457|	Collagen alpha-1(I) chain
*WIPI1*^#^	0.87	sp|Q5MNZ9|	WD repeat domain phosphoinositide-interacting protein 1
*ARMC3*^#^	0.88	sp|Q5W041|	Armadillo repeat-containing protein 3
*CEA20*^#^	0.89	sp|Q6UY09|	Carcinoembryonic antigen-related cell adhesion molecule 20
*GLIS3*^#^	0.75	sp|Q8NEA6|	Zinc finger protein GLIS3
*SRSF4*^#^	0.79	sp|Q8VE97|	Serine/arginine-rich splicing factor 4
*LAMC3*^#^	0.82	sp|Q9Y6N6|	Laminin subunit gamma-3
*TNNI1*^#^*	0.96	sp|P02645|	Troponin I, slow skeletal muscle
TNNT2*	0.81	sp|P02642|	Cardiac muscle isoforms
TNNC1*	0.70	sp|P09860|	Troponin C, slow skeletal and cardiac muscles
MYOZ1*	0.76	sp|Q8SQ24|	Myozenin-1
MYBPH*	0.72	sp|Q05623|	Myosin-binding protein H
COEA1*	0.87	sp|P32018|	Collagen alpha-1(XIV) chain
KCRS*	0.82	sp| P11009|	Creatine kinase S-type, mitochondrial
DEP1A*	0.75	sp| Q5TB30|	DEP domain-containing protein 1A

^#^Genes selected from the 43 DEGs shared by all time points and both Gaoyou and Jinding duck breeds;

*genes selected from the 393 DEGs with expression patterns in line with the muscle development curve in both Gaoyou and Jinding duck breeds

### Functional annotation of DEGs

**Fig 4:**
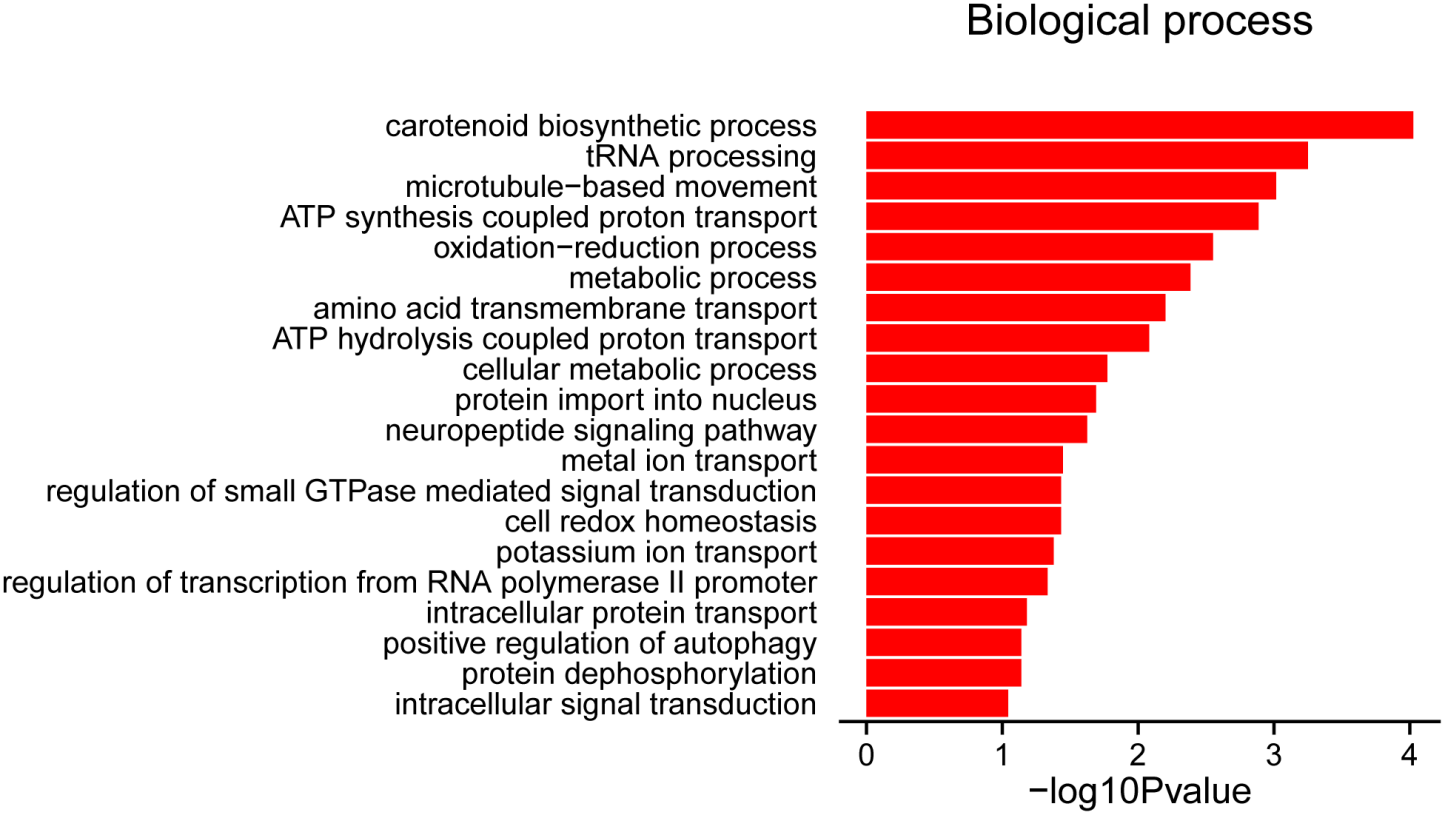
Significantly enriched gene ontology terms (*P*<0.1, hit list ≥3) among 393 differentially expressed genes in Gaoyou and Jinding duck breeds. All GO terms belong to ‘biological processes’.

All 393 DEGs in this study were mapped to terms in the gene ontology (GO) database to determine their function, and GO term enrichment analysis (*P*<0.1, hit list ≥3) was performed. A total of 137 genes were categorized in only one GO classification category (BP, biological process), and no DEGs were categorized in the cellular components (CC) or molecular functions (MF) categories. The top BP terms were: GO0016117 (carotenoid biosynthetic process), followed by GO0008033 (tRNA processing), GO0007018 (microtubule-based movement), and GO0015986 (ATP-synthesis-coupled proton transport) (Fig 4 and S6 Table).

### Pathways and regulatory network for muscle development in ducks

**Fig 5:**
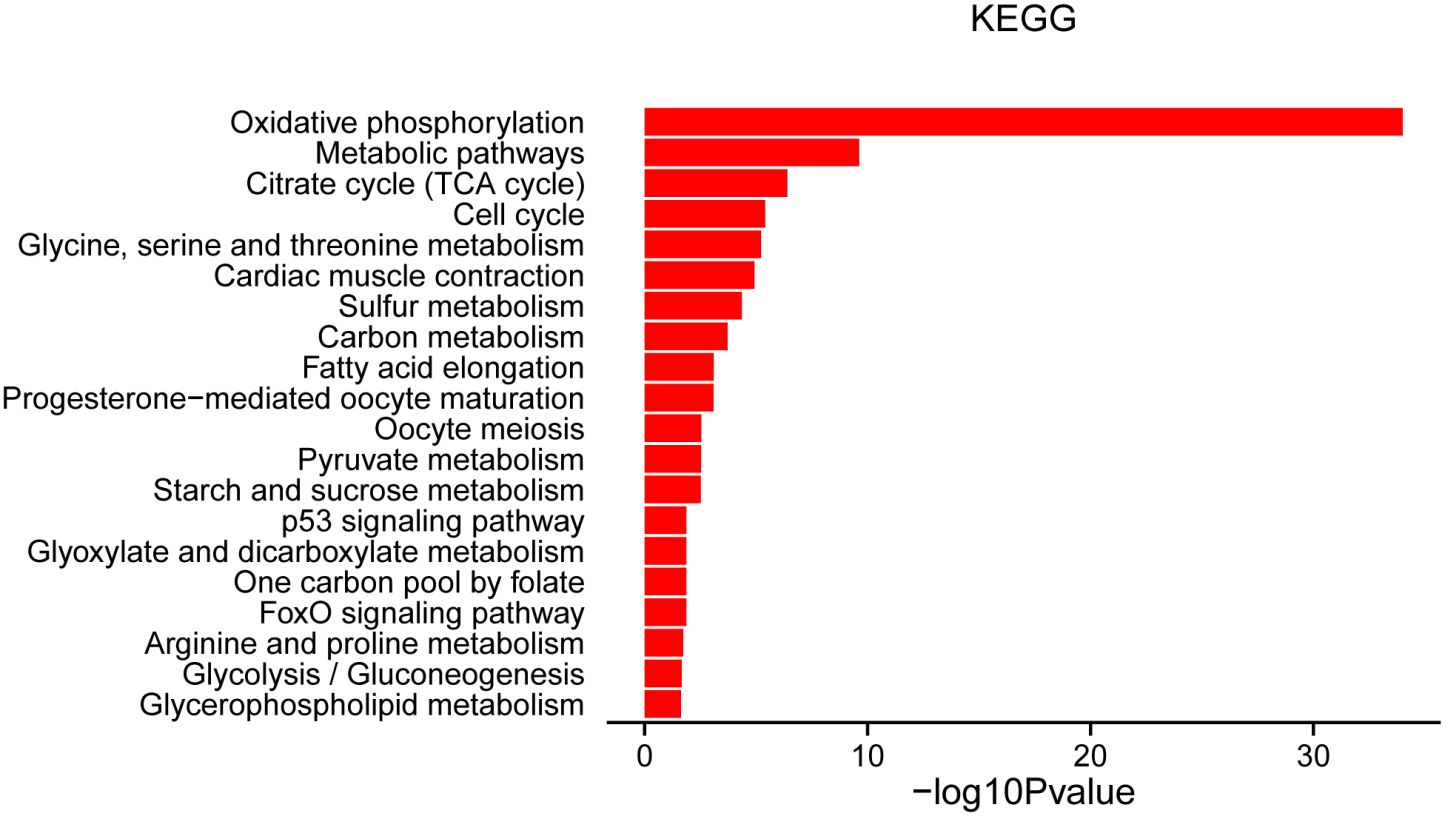
Significantly enriched KEGG pathways (*P*<0.05, hit list ≥3) among 393 differentially expressed genes in Gaoyou and Jinding duck breeds.

The regulation of muscle development may entail complex pathway interactions involving muscle, fat, and connective tissue, necessitating the analysis of regulatory networks. KEGG pathway analysis of the known 393 DEGs related to muscle development and lipid metabolism identified 24 pathways (*P*<0.05, hit list >3) (Fig 5). Well-known pathways affecting metabolism were enriched, including metabolic pathways, citrate cycle (TCA cycle), glycine, serine, and threonine metabolism, sulfur metabolism, carbon metabolism, and pyruvate metabolism. Pathways affecting lipid metabolism included fatty acid elongation, glycine, serine, and threonine metabolism, and arginine and proline metabolism (Fig 5 and S7 Table).

### Functional annotation of DEGs within breeds among different developmental periods

The top 20 GO terms and pathways in the two duck breeds at different developmental periods are shown in S5 Table. The two breeds shared several GO terms and pathways in the same development periods, while each breed also had distinct GO terms and pathways. Similar results were found for the other analyzed period (E27 vs. 5 dph).

## Discussion

We previously found that PM mass stopped increasing from E21 to E25, and actually decreased before hatching, then increasing from hatching to 7 dph in both Gaoyou and Jinding duck breeds. The PM mass in Gaoyou ducks was significantly higher than in Jinding ducks during the early development period (S1 Fig)[4]. We also analyzed the characteristics (CSA, diameter) of PM in the above two duck breeds and found results that were consistent with our earlier report[1]. This phenomenon was also reported in turkeys by Moore et al.[14], who found that the CSA of myofibers and satellite cell mitotic activity in PM decreased in late-term turkey embryos. We investigated the genes and molecular mechanisms involved in this developmental process by carrying out a comprehensive analysis of genes with expression levels that reflected the PM growth pattern. (i.e., genes with higher or lower expression levels at E27 compared with E21 and 5 dph) in Gaoyou and Jinding ducks. We found that 43 DEGs, including 19 novel genes, were shared at all time points and by both duck breeds. Furthermore, 393 DEGs had expression patterns in line with the PM growth patterns, among which 204 genes showed lower expression at E27 and 189 genes showed higher expression levels. The RNA-Seq data were confirmed to be reliable by *q*RT-PCR. These identified DEGs included many genes significantly related to muscle development.

### DEGs shared by both duck breeds at all time points

Forty-three genes (including 19 novel genes) were differentially expressed in both duck breeds at all analyzed time points. These genes may functionally influence the developmental of PM in duck breeds during the late-term embryonic to neonatal periods. Fructose-1,6-bisphosphatase 1 (*F16P1*) is a key regulatory enzyme of gluconeogenesis. It plays an essential role in metabolism and development in most organisms[15], and is strongly regulated by Ca^2+^[16]. *F16P1* gene expression decreased steadily from E21 to 5 dph, with significantly lower levels in Jinding compared with Gaoyou ducks. This change may reflect insufficient nutrition, especially in terms of Ca^2+^. Avian embryos use their limited energy reserves (yolk) to meet the demand of hatching activities, but the yolk reserves decrease during embryonic development and absorption was defective in early neonatal period.

Collagen alpha-1(I) chain (*COL1A1*), laminin subunit gamma-3 (*LAMC3)*, and FSD1-like protein (*FSD1L*) are important components of the basement membrane that act as a supporting scaffold and fix the epithelial cells to the connective tissue. The expression level of *COL1A1* was higher than *LAMC3* and *FSD1L*, with the lowest expression level occurring at E27 compared with E21 and 5 dph, and significantly lower expression in Jinding compared with Gaoyou ducks. Collagen is one of the main components of the endomysium and perimysium. High expression of *COL1A1* observed in other animals during this developmental period suggests that birds require additional energy at this time to fix the connective tissue and support the subsequent development of muscle cells. *COL1A1* gene expression levels are correlated with age and meat taste[17], suggesting that it may influence the quantity and quality of duck PM. *TNNI1* was also differentially expressed in both duck breeds at all analyzed time points, as discussed below.

### DEGs with lower expression at E27

The troponin complex in skeletal and cardiac muscles, which plays a central role in Ca^2+^-regulated contraction, consists of three subunits: a Ca^2+^-binding subunit (troponin C, *TNNC*); an inhibitory subunit (troponin I, *TNNI*); and a tropomysin-binding subunit (troponin T, *TNNT*)[18], which are required for normal growth and breathing, and are essential for postnatal survival[19,20]. In the current study, *TNNT2* (cardiac muscle isoform), *TNNI1* (slow skeletal muscle isoform), and *TNNC1* 3 (slow skeletal and cardiac muscles isoform) showed the lowest expression levels at E27, and expression levels up to 443.98 times higher at E21 in Jinding ducks. During the embryonic period, expression of the TNNT isoforms was not restricted to skeletal and cardiac muscle fiber types[21,22], and the complex expression and alternative splicing pattern of *TNNT2* in embryonic and neonatal skeletal muscles indicated that it was regulated by a genetically programmed biological clock, rather than by adaptation to changes in contractile function[18]. *TNNI* has evolved into *TNNI1*, *TNNI2*, and *TNNI3* isoforms, which are expressed under muscle-type-specific and developmental regulation[23]. *TNNI1* and *TNNI2* genes are switched on during skeletal muscle myogenesis and are co-expressed during the early periods of development, later becoming restricted to slow and fast skeletal muscle fibers, respectively[24,25]. Ma et al.[26] reported that an increase in intramuscular fat (IMF) content in pigs was positively correlated with the increased abundance of slow-twitch troponin I (TNNI1) protein and negatively correlated with myosin heavy chain IIb (MyHCIIb) protein content. It has been suggested that proteome changes in longissimus muscle contributed to the increased IMF content in L-arginine-supplemented pigs[26]. The *TNNC1* gene is expressed in both slow skeletal (*sTNNC*) and cardiac tissues (*cTNNC*). During development, both *cTNNC* and *sTNNC* are expressed in embryonic skeletal muscle, but expression of the *cTNNC* gene is subsequently turned off during the transition to fast-twitch muscle[27]. IGFs are synthesized in tissues and play an important role in embryonic development in birds via autocrine/paracrine mechanisms[28]. Higher IGF1 expression levels were found in muscle from E15-E18 with a peak at E17 followed by a decline during chick embryonic development[28]. We previously reported that *IGF1* and *MSTN* mRNA levels may influence muscle-growth rates in ducks[3]. *IGF1* positively regulates muscle cell differentiation, and affects gene and microRNA expression in this process[29]. In this study, *IGF1* expression was lower at E27 compared with E21 and 5 dph in both duck breeds. This expression pattern and level were consistent with that reported by Hu et al.[3], and indicated that IGF1 may play an important role in PM development in ducks. IGF2BP3 (IGF2 mRNA-binding protein 3), as a member of the IGF2 mRNA-binding protein family, is expressed mainly during embryonic development[30]. IGF2BP3 plays a critical role in regulating cell proliferation via an IGF2-dependent pathway in K562 leukemia cells[30]. IGF2 is also a major fetal growth factor, and similar to IGF2, IGF2 mRNA-binding proteins are mainly expressed in developing epithelia, muscle, and placenta in both mouse and human embryos[31]. Irisin, a novel myokine encoded by the *FNDC5* gene, is reported to stimulate brown fat-like development of white fat tissue and thermo genesis in mammals, which is highly related to metabolic activity in skeletal muscle and brown fat[32,33]. At baseline, local expression of *FNDC5* in skeletal muscle is associated with mRNA expression of *IGF1* and mitochondrial function and mitochondria-related gene expression (such as *PPARγ*) in obese subjects with reduced growth hormone, suggesting a potential role for local-acting *FNDC5* in muscle in a low-growth hormone state[34]. In our study, *FNDC5* and *IGF1* gene expression patterns were similar, and lowest at E27. Other genes with reduced expression at E27, such as *CENPF*[35] and *SCOT1*[36], were also correlated with the development of PM and fatty deposits. *CENPF* is highly homologous to other cell cycle regulatory proteins such as mitosin and CMF1, *CMF1* regulates myocyte differentiation by interaction with Rb family members to induce the expression of myogenic regulatory factors, suggesting its involvement in regulating the cell cycle[35,37].

### DEGs with higher expression at E27

*MUSTN1* is involved in myogenic fusion and differentiation in various animals, and is an essential regulator of myogenic differentiation and myofusion. Its downstream targets are *MyoD1* and *MyoG*[38]. The relative growth rates of breast and leg muscles in Beijing ducks were correlated with the expression levels of the *MUSTN1* gene[39]. In the current study, PM development was slowest or even absent at E27, when *MUSTN1* gene expression increased rapidly to its highest level (>6-fold in Gaoyou ducks and 5.14-fold in Jinding ducks) compared with E21, and the expression level was down-regulated again (about 0.31-fold in Gaoyou and 0.48-fold in Jinding ducks) at 5 dph, when PM weight increased again. These results were consistent with the above-mentioned report, and suggest that *MUSTN1* is involved in the growth of breast muscle. Muscle specification and differentiation also require muscle regulatory factors (MRFs), including *MYOD1*, *MyoG*, *Myf6*, *and Myf5*, which are required for commitment of proliferating somatic cells to the myogenic linage and increase the transcriptional activation of various downstream target genes. In this study, *MYOD1* gene expression followed the same pattern as *MUSTN1* in both duck breeds, with the highest expression levels at E27. The expression patterns of the *MUSTN1* and *MYOD1* genes at the studied time points indicated that myogenic differentiation and myofusion were inhibited. We assessed *MYOD1* gene functioning by gene silencing in duck primary embryonic myoblasts and showed that down-regulated expression of *MYOD1* induced cell proliferation and inhibited differentiation, accompanied by a >2-fold down-regulation of *MyoG*, *Myf6*, and *MSTN* (*TGF-beta* super-family member and a potent negative regulator of skeletal muscle growth) expression and up-regulation of *Myf5* expression (data not published). These results revealed the important role of *MYOD1* in duck embryonic myoblasts.

The *MYOZ1* gene-encoded calsarcin-2 protein is expressed exclusively in fast-twitch muscles[40]. Mice deficient in calsarcin-2 have substantially reduced body weights and fast-twitch muscle mass in the absence of an overt myopathic phenotype[41]. The swine *MYOZ1* promoter improved the IMF content and influenced the quality of meat in pigs by up-regulating *sPPAR-γ2* (a key regulator of adipocyte differentiation) expression in skeletal muscle[40]. The higher expression level of the *MYOZ1* gene at E27 in the current study may indicate that the composition of skeletal muscle, in terms of the relative numbers of slow- and fast-twitch fibers, could be adjusted during this development stage. Synemin is necessary for maintaining membrane integrity and regulating signaling molecules during muscle hypertrophy[42–44]. The absence of synemin results in decreased fiber size and increased sarcolemmal deformability and susceptibility to injury[45], and is encoded by the *SYNEM* gene, which showed higher expression at E27 in both duck breeds Other genes that showed higher expression levels at E27 included *ADIPO* (adiponectin, which might slightly enhanced adipogenesis in skeletal muscle and is associated with the intramuscular fat content)[46,47], *MYRIP* (Rab effector MyRIP, which belongs to the protein kinase A (PKA)-anchoring family, is implicated in hormone secretion, functions as a scaffolding protein that links PKA to components of the exocytosis machinery)[48], *MYBPH* (myosin-binding protein H, which is conserved in various animals including chickens, and is a component of the thick filaments of the skeletal muscle and has strong affinity for myosin)[49,50].

### Go and KEGG pathways

Only the BP GO category was categorized in this study. All genes involved in the GO term 0007018 microtubule-based movement (*P<0*.*001*) (kinesin-like proteins *KIF11*, *KIF14*, *KIF15*, *KIF20*, *KIF23* and centromere-associated protein E, *CENPE)* showed lower expression levels at E27 compared with E21 and 5 dph. Kinesin-family proteins are a class of microtubule-based motor proteins that function in mitosis as well as meiosis in both plant and animal systems[51,52]. Similarly, all genes included in the GO term 0006606 protein import to nucleus (centromere protein F *CENPF*, *CEP89*, *IMA2*, *HMMR*, *KIF15*) showed decreased expression levels at E27 compared with E21 and 5 dph, while all genes included in the GO term 0006470, protein dephosphorylation, were highly expressed at E27 compared with E21 and 5 dph.

Pathways affecting lipid metabolism were identified in this study, including fatty acid elongation. All three genes in this pathway (*MECR*, *ACOT1*, and *ELOVL1*) were down-regulated in E27. The cytosolic form of MECR (cMECR) is expressed in the cytosol and/or nuclear region, and binds directly to PPARα and enhances PPARα activity[53]. Elongation of very-long-chain fatty acids 1 (*ELOVL1*) is an ubiquitously expressed gene, the product of which was thought to be associated with elongation of carbon chains in fatty acids[54]. Well-known metabolic pathways (such as citrate cycle and glycine, serine, and threonine metabolism) were enriched in the DEGs identified in this study, with most genes involved in these pathways showing higher expression levels at E27. During development, the metabolic needs of skeletal muscle can change to adapt to its functional characteristics, and our results suggest that the metabolic demands were increased when muscle development was reduced at E27. In contrast, all the genes involved in the cell cycle pathway showed lower expression levels at E27 compared with E21 and 5 dph. Cell cycle arrest is known to be a prerequisite for myoblast fusion and subsequent differentiation[55]. In this study, the growth of breast muscle in both duck breeds was static or decreased at E27, while the genes involved in the cell cycle were inhibited and their expression levels were reduced. These results indicated that these cells either entered cell cycle, or showed cell cycle arrest during myoblast cell fusion or differentiation, consistent with the above report[55]. Most genes (e.g., various cytochrome c oxidase and cytochrome b-c1 complex subunits) involved with the pathway cardiac muscle contraction showed higher expression levels at E27, except for *TNNT2* and *TNNC1* which showed lower expression levels. These results may indicate that, during this static growth period, breast muscle function is maintained, with activated metabolic pathways and arrested cell cycle.

Although some genes were differentially expressed between duck breeds at different development periods, comparisons of E21 vs. E27, and E27 vs. 5 dph within each breed showed that >50% of DEGs were shared by both breeds, and >76.74% were expressed during late embryonic development (E21 vs E27). The effect of duck breed on DEGs was therefore less than that of age. The 393 DEGs shared by different periods and both duck breeds may help to reveal the mechanism of PM development during the late embryonic period and early post-hatching. We also found more DEGs in E21 vs. E27 compared with E27 vs. 5 dph, suggesting that more changes occurred during the late embryonic period. This could be because the organs and tissues were becoming prepared to survive in a changeable environment after hatching. GO and pathway analyses of these DEGs showed the presence of distinct as well as shared terms and pathways between different breeds, indicating that PM development in ducks may be controlled by similar mechanisms in different breeds (as noted above), while other mechanisms may be specific to different breeds. The GO terms shared between E21 and E27 included both BP and MF terms, but not CC terms, while three CC terms, two MF terms, and one BP term were shared between E27 and 5 dph. The DEGs detected in different developmental periods were involved in different functional annotations. Pathway analysis revealed 9 and 10 pathways shared in both breeds at E21 vs. E27 and E27 vs. 5 dph, respectively, most of which were involved in metabolism, such as oxidative phosphorylation, the citrate cycle (TCA cycle), carbon metabolism, and other amino acid metabolism. DEGs involved in ECM–receptor interactions, especially collagen family members, also play an important role in affecting muscle development [56].

**In conclusion,** about 393 genes were differentially expressed at the analyzed time points, which was in accord with the curve of PM development during the early developmental period. Forty-three DEGs were shared across all the analyzed time points and both duck breeds. We discussed the functions of 19 genes (summarized in S8 Table) that may be important candidate genes involved in duck PM development. More than 50% of DEGs (detected in E21 vs. E27 and E27 vs. 5 dph) were shared by both breeds, suggesting that breed may have less effect on PM development than developmental age. We also identified DEGs involved in different metabolism and cell cycle pathways that may functionally influence PM development. These results provide valuable information regarding the mechanisms underlying PM development in ducks.

## Supporting information

S1 Table Basic statistics for RNA-seq reads generated from pectoralis muscle of Gaoyou and Jinding ducks. (XLSX)

S2 Table Selected qRT-PCR primer sequences. *ACTB and *GAPDH* were the endogenous control genes.
(XLSX)

S3 Table Differentially expressed genes shared by both Gaoyou and Jinding duck breeds among three developmental time points (embryonic days 21 and 27 and post-hatching day 5), including 19 novel genes. (XLSX)

S4 Table List of 393 differentially expressed genes with expression patterns in line with the muscle development curve in both Gaoyou and Jinding duck breeds. Sheet 1, RPKM for 393 DEGs; sheet 2, fold changes for 393 DEGs.
(XLSX)

S5 Table List of DEGs within duck breeds at different time periods (E21 vs. E27 and E27 vs. 5 dph), and top 20 enriched GO terms and pathways among DEGs within Gaoyou and Jinding ducks at different time periods. Sheets 1–4: Lists of DEGs within Gaoyou and Jinding ducks at E21 vs. E27 and E27 vs. 5 dph; sheet 5: DEGs within Gaoyou and Jinding ducks; sheet 6: top 20 GO terms; sheet 7: top 20 pathways.
(XLSX)

S6 Table Enriched gene ontology terms among 393 differentially expressed genes in Gaoyou and Jinding duck breeds. All GO terms belong to ‘biological processes’. Sheet 1, all enriched GO terms; sheet 2, significant GO terms (*P*<0.1, hit list ≥3); sheet 3, details of different significant GO terms.
(XLSX)

S7 Table Enriched KEGG pathways among 393 differentially expressed genes in Gaoyou and Jinding duck breeds. Sheet 1, all enriched KEGG pathways; sheet 2, significant KEGG pathways (*P*<0.05, hit lists ≥3); sheet 3, details of different significant KEGG pathways.
(XLSX)

S8 Table Gene list summarized in Discussion. (XLSX)

S1 Fig Profiles of body weight (left) and PM weight (right) during early development in ducks. (DOCX)

## References

[1] Li HF, Shu JT, Shan YJ, Chen WF, Song C, Xu WJ. Myofiber development during embryonic to neonatal development in duck breeds differing in muscle growth rates. Journal of Integrative Agriculture. 2016; 15: 403–413.

[2] Shu JT, Li HF, Shan Y, Xu WJ, Chen W, Song C, et al Expression profile of IGF-I-calcineurin-NFATc3-dependent pathway genes in skeletal muscle during early development between duck breeds differing in growth rates. Dev Genes Evol. 2015; 225: 139–148. doi: 10.1007/s00427-015-0501-8 25963597 10.1007/s00427-015-0501-8

[3] Hu Y, Liu HX, Song C, Xu WJ, Ji GG, Zhu CH, et al Profiles of mRNA expression of related genes in the duck hypothalamus-pituitary growth axis during embryonic and early post-hatch development. Gene. 2015; 559: 38–43. doi: 10.1016/j.gene.2015.01.009 25577952 10.1016/j.gene.2015.01.009

[4] Hu Y, Liu HX, Shan YJ, Ji GG, Xu WJ, Shu JT, et al The relative expression levels of insulin-like growth factor 1 and myostatin mRNA in the asynchronous development of skeletal muscle in ducks during early development. Gene. 2015; 567: 235–243. doi: 10.1016/j.gene.2015.05.001 25943637 10.1016/j.gene.2015.05.001

[5] Jing DL, Zhang QQ, Cen LS, Xie DJ, Liang XN. Study of embryonic growth and development of organs in Duck. Jiangsu Agric. Sci. 2007; 1: 122–124 (in Chinese)

[6] Chen W, Tangara M, Xu J, Peng J. Developmental transition of pectoralis muscle from atrophy in late-term duck embryos to hypertrophy in neonates. Exp Physiol. 2012; 97: 861–872. doi: 10.1113/expphysiol.2011.01083.x 22787243 10.1113/expphysiol.2011.01083.x

[7] Picard B, Lefaucheur L, Berri C, Duclos MJ. Muscle fibre ontogenesis in farm animal species. Reproduction Nutrition Development. 2002; 42: 415–431. 10.1051/rnd:2002035 12537254

[8] Trapnell C, Roberts A, Goff L, Pertea G, Kim D, Kelley DR, et al Differential gene and transcript expression analysis of RNA-seq experiments with TopHat and Cufflinks. Nat Protoc. 2012; 7: 562–578. doi: 10.1038/nprot.2012.016 22383036 10.1038/nprot.2012.016 PMC3334321

[9] Huang Y, Li Y, Burt DW, Chen H, Zhang Y, Qian W, et al The duck genome and transcriptome provide insight into an avian influenza virus reservoir species. Nature Genetics. 2013; 45: 776–783. doi: 10.1038/ng.2657 23749191 10.1038/ng.2657 PMC4003391

[10] Chen L, Luo J, Li JX, Li JJ, Wang DQ, Tian Y, et al Transcriptome analysis of adiposity in domestic ducks by transcriptomic comparison with their wild counterparts. Animal Genetics. 2015; 46: 299–307. doi: 10.1111/age.12294 25917302 10.1111/age.12294

[11] Xu T, Gu L, Schachtschneider KM, Liu X, Huang W, Xie M, et al Identification of differentially expressed genes in breast muscle and skin fat of postnatal Pekin duck. PLoS One. 2014; 9: e107574 doi: 10.1371/journal.pone.0107574 25264787 10.1371/journal.pone.0107574 PMC4180276

[12] Mortazavi A, Williams BA, McCue K, Schaeffer L, Wold B. Mapping and quantifying mammalian transcriptomes by RNA-Seq. Nature Methods. 2008; 5: 621–628. doi: 10.1038/nmeth.1226 18516045 10.1038/nmeth.1226 PMC13303166

[13] Benjamini Y, Hochberg Y. Controlling the false discovery rate: a practical and powerful approach to multiple testing. Journal of the Royal Statistical Society. Series B (Methodological). 1995: 289–300.

[14] Moore DT, Ferket PR, Mozdziak PE. Muscle development in the late embryonic and early post-hatch poult. International Journal of Poultry Science. 2005; 4:138–142.

[15] Gizak A, Rakus D, Dzugaj A. Nuclear localization of fructose 1,6-bisphosphatase in smooth muscle cells. J Mol Histol. 2005; 36: 243–248. doi: 10.1007/s10735-005-6523-1 16200456 10.1007/s10735-005-6523-1

[16] Gizak A, Majkowski M, Dus D, Dzugaj A. Calcium inhibits muscle FBPase and affects its intracellular localization in cardiomyocytes. FEBS Letters. 2004; 576: 445–448. doi: 10.1016/j.febslet.2004.09.050 15498578 10.1016/j.febslet.2004.09.050

[17] Piorkowska K, Zukowski K, Nowak J, Poltowicz K, Ropka-Molik K, Gurgul A. Genome-wide RNA-Seq analysis of breast muscles of two broiler chicken groups differing in shear force. Animal Genetics. 2016; 47: 68–80. doi: 10.1111/age.12388 26592359 10.1111/age.12388

[18] Jin JP. Alternative RNA splicing-generated cardiac troponin T isoform switching: a non-heart-restricted genetic programming synchronized in developing cardiac and skeletal muscles. Biochemical & Biophysical Research Communications. 1996; 225: 883–889. 8780706 10.1006/bbrc.1996.1267

[19] Ju Y, Li J, Xie C, Ritchlin CT, Xing L, Hilton MJ, et al Troponin T3 expression in skeletal and smooth muscle is required for growth and postnatal survival: characterization of Tnnt3(tm2a(KOMP)Wtsi) mice. Genesis. 2013; 51: 667–675. doi: 10.1002/dvg.22407 23775847 10.1002/dvg.22407 PMC3787964

[20] Clause KC, Tchao J, Powell MC, Liu LJ, Huard J, Keller BB, et al Developing cardiac and skeletal muscle share fast-skeletal myosin heavy chain and cardiac troponin-I expression. PLoS One. 2012; 7: e40725 doi: 10.1371/journal.pone.0040725 22808244 10.1371/journal.pone.0040725 PMC3393685

[21] Jin J-P. Evolution, regulation, and function of N-terminal variable region of troponin T: Modulation of muscle contractility and beyond. Int Rev Cell Mol Biol. 2016; 321: 1–28. doi: 10.1016/bs.ircmb.2015.09.002 26811285 10.1016/bs.ircmb.2015.09.002

[22] Wei B, Jin J-P. TNNT1, TNNT2, and TNNT3: Isoform genes, regulation, and structure-function relationships. Gene. 2016; 582: 1–13. doi: 10.1016/j.gene.2016.01.006 26774798 10.1016/j.gene.2016.01.006 PMC5325693

[23] Sheng J-J, Jin J-P. TNNI1, TNNI2 and TNNI3: Evolution, regulation, and protein structure-function relationships. Gene. 2016; 576: 385–394. doi: 10.1016/j.gene.2015.10.052 26526134 10.1016/j.gene.2015.10.052 PMC5798203

[24] Yang H, Xu ZY, Lei MG, Li FE, Deng CY, Xiong YZ, et al Real-time reverse transcription-PCR expression profiling of porcine troponin I family in three different types of muscles during development. Molecular Biology Reports. 2011; 38: 827–832. doi: 10.1007/s11033-010-0172-5 20376701 10.1007/s11033-010-0172-5

[25] Mullen AJ, Barton PJ. Structural characterization of the human fast skeletal muscle troponin I gene (TNNI2). Gene. 2000; 242: 313–320. 10721725 10.1016/s0378-1119(99)00519-3

[26] Ma X, Zheng C, Hu Y, Wang L, Yang X, Jiang Z. Dietary L-arginine supplementation affects the skeletal longissimus muscle proteome in finishing pigs. PLoS One. 2015; 10: e0117294 doi: 10.1371/journal.pone.0117294 25635834 10.1371/journal.pone.0117294 PMC4311982

[27] Li MX, Hwang PM. Structure and function of cardiac troponin C (TNNC1): Implications for heart failure, cardiomyopathies, and troponin modulating drugs. Gene. 2015; 571: 153–166. doi: 10.1016/j.gene.2015.07.074 26232335 10.1016/j.gene.2015.07.074 PMC4567495

[28] Liu YL, Guo W, Pu ZY, Li XY, Lei XY, Yao JH, et al Developmental changes of insulin-like growth factors in the liver and muscle of chick embryos. Poult Sci. 2016; 95: 1396–1402. doi: 10.3382/ps/pew043 26944971 10.3382/ps/pew043

[29] Su R, Sun W, Li D, Wang QZ, Lv XY, Musa HH, et al Association between DLK1 and IGF-I gene expression and meat quality in sheep. Genetics and Molecular Research: GMR. 2014; 13: 10308–10319. doi: 10.4238/2014.December.4.26 25501243 10.4238/2014.December.4.26

[30] Liao B, Hu Y, Herrick DJ, Brewer G. The RNA-binding protein IMP-3 is a translational activator of insulin-like growth factor II leader-3 mRNA during proliferation of human K562 leukemia cells. The Journal of Biological Chemistry. 2005; 280: 18517–18524. doi: 10.1074/jbc.M500270200 15753088 10.1074/jbc.M500270200

[31] Nielsen J, Christiansen J, Lykke-Andersen J, Johnsen AH, Wewer UM, Nielsen FC. A family of insulin-like growth factor II mRNA-binding proteins represses translation in late development. Molecular and Cellular Biology. 1999; 19: 1262–1270. 9891060 10.1128/mcb.19.2.1262 PMC116055

[32] Li X, Fang W, Hu Y, Wang Y, Li J. Characterization of fibronectin type III domain-containing protein 5 (FNDC5) gene in chickens: Cloning, tissue expression, and regulation of its expression in the muscle by fasting and cold exposure. Gene. 2015; 570: 221–229. doi: 10.1016/j.gene.2015.06.022 26072164 10.1016/j.gene.2015.06.022

[33] Rabiee F, Forouzanfar M, Ghazvini Zadegan F, Tanhaei S, Ghaedi K, Motovali Bashi M, et al Induced expression of Fndc5 significantly increased cardiomyocyte differentiation rate of mouse embryonic stem cells. Gene. 2014; 551: 127–137. doi: 10.1016/j.gene.2014.08.045 25168892 10.1016/j.gene.2014.08.045

[34] Srinivasa S, Suresh C, Mottla J, Hamarneh SR, Irazoqui JE, Frontera W, et al FNDC5 relates to skeletal muscle IGF-I and mitochondrial function and gene expression in obese men with reduced growth hormone. Growth Horm IGF Res. 2016; 26: 36–41. doi: 10.1016/j.ghir.2015.12.008 26774404 10.1016/j.ghir.2015.12.008 PMC4716612

[35] Dees E, Miller PM, Moynihan KL, Pooley RD, Hunt RP, Galindo CL, et al Cardiac-specific deletion of the microtubule-binding protein CENP-F causes dilated cardiomyopathy. Dis Model Mech. 2012; 5: 468–480. doi: 10.1242/dmm.008680 22563055 10.1242/dmm.008680 PMC3380710

[36] Shafqat N, Kavanagh KL, Sass JO, Christensen E, Fukao T, Lee WH, et al A structural mapping of mutations causing succinyl-CoA:3-ketoacid CoA transferase (SCOT) deficiency. J Inherit Metab Dis. 2013; 36: 983–987. doi: 10.1007/s10545-013-9589-z 23420214 10.1007/s10545-013-9589-z PMC3825524

[37] Robertson JB, Zhu T, Nasreen S, Kilkenny D, Bader D, Dees E. CMF1-Rb interaction promotes myogenesis in avian skeletal myoblasts. Dev Dyn. 2008; 237: 1424–1433. doi: 10.1002/dvdy.21544 18425850 10.1002/dvdy.21544 PMC2854498

[38] Liu C, Gersch RP, Hawke TJ, Hadjiargyrou M. Silencing of Mustn1 inhibits myogenic fusion and differentiation. Am J Physiol Cell Physiol. 2010; 298: C1100–8. doi: 10.1152/ajpcell.00553.2009 20130207 10.1152/ajpcell.00553.2009 PMC2867393

[39] Xu TS, Gu LH, Sun Y, Zhang XH, Ye BG, Liu XL, et al Characterization of MUSTN1 gene and its relationship with skeletal muscle development at postnatal stages in Pekin ducks. Genetics and Molecular Research: GMR. 2015; 14: 4448–4460. doi: 10.4238/2015.May.4.2 25966217 10.4238/2015.May.4.2

[40] Ma J, Chai J, Shang Y, Li Y, Chen R, Jia J, et al Swine PPAR-γ2 expression upregulated in skeletal muscle of transgenic mice via the swine Myozenin-1 gene promoter. Transgenic Research. 2015; 24: 409–420. doi: 10.1007/s11248-014-9849-1 25421932 10.1007/s11248-014-9849-1

[41] Frey N, Frank D, Lippl S, Kuhn C, Kogler H, Barrientos T, et al Calsarcin-2 deficiency increases exercise capacity in mice through calcineurin/NFAT activation. The Journal of Clinical Investigation. 2008; 118: 3598–3608. doi: 10.1172/JCI36277 18846255 10.1172/JCI36277 PMC2564612

[42] Mizuno Y, Guyon JR, Okamoto K, Kunkel LM. Expression of synemin in the mouse spinal cord. Muscle & Nerve. 2009; 39: 634–641. doi: 10.1002/mus.21221 19229966 10.1002/mus.21221 PMC2868828

[43] Li Z, Parlakian A, Coletti D, Alonso-Martin S, Hourde C, Joanne P, et al Synemin acts as a regulator of signalling molecules during skeletal muscle hypertrophy. Journal of Cell Science. 2014; 127: 4589–4601. doi: 10.1242/jcs.143164 25179606 10.1242/jcs.143164

[44] Paul M, Skalli O. Synemin: Molecular features and the use of proximity ligation assay to study its interactions. Methods Enzymol. 2016; 568: 537–555. doi: 10.1016/bs.mie.2015.08.005 26795483 10.1016/bs.mie.2015.08.005

[45] Garcia-Pelagio KP, Muriel J, O’Neill A, Desmond PF, Lovering RM, Lund L, et al Myopathic changes in murine skeletal muscle lacking synemin. Am J Physiol Cell Physiol. 2015; 308: C448–62. doi: 10.1152/ajpcell.00331.2014 25567810 10.1152/ajpcell.00331.2014 PMC4360028

[46] Martins TS, Sanglard LMP, Silva W, Chizzotti ML, Renno LN, Serao NVL, et al Molecular factors underlying the deposition of intramuscular fat and collagen in skeletal muscle of nellore and Angus Cattle. PLoS One. 2015; 10: e0139943 doi: 10.1371/journal.pone.0139943 26436893 10.1371/journal.pone.0139943 PMC4593631

[47] Indrakusuma I, Sell H, Eckel J. Novel mediators of adipose tissue and muscle crosstalk. Curr Obes Rep. 2015; 4: 411–417. doi: 10.1007/s13679-015-0174-7 26349436 10.1007/s13679-015-0174-7

[48] Brozzi F, Lajus S, Diraison F, Rajatileka S, Hayward K, Regazzi R, et al MyRIP interaction with MyoVa on secretory granules is controlled by the cAMP-PKA pathway. Mol Biol Cell. 2012; 23: 4444–4455. doi: 10.1091/mbc.E12-05-0369 22993210 10.1091/mbc.E12-05-0369 PMC3496617

[49] Jung J, Oh J, Lee K. Nucleotide and deduced amino acid sequences of rat myosin binding protein H (MyBP-H). Arch Pharm Res. 1998; 21: 712–717. 9868543 10.1007/BF02976763

[50] Conti A, Riva N, Pesca M, Iannaccone S, Cannistraci CV, Corbo M, et al Increased expression of Myosin binding protein H in the skeletal muscle of amyotrophic lateral sclerosis patients. Biochimica et Biophysica Acta. 2014; 1842: 99–106. doi: 10.1016/j.bbadis.2013.10.013 24184715 10.1016/j.bbadis.2013.10.013

[51] Goodson HV, Kang SJ, Endow SA. Molecular phylogeny of the kinesin family of microtubule motor proteins. Journal of Cell Science. 1994; 107 (Pt 7): 1875–1884. 7983154 10.1242/jcs.107.7.1875

[52] Li J, Yan S, Yang W, Li Y, Xia M, Chen Z, et al Transcriptomic analysis reveals the roles of microtubule-related genes and transcription factors in fruit length regulation in cucumber (Cucumis sativus L.). Scientific Reports. 2015; 5: 8031 doi: 10.1038/srep08031 25619948 10.1038/srep08031 PMC5379036

[53] Kim D-G, Yoo JC, Kim E, Lee Y-S, Yarishkin OV, Lee DY, et al A novel cytosolic isoform of mitochondrial trans-2-enoyl-CoA reductase enhances peroxisome proliferator-activated receptor α activity. Endocrinol Metab (Seoul). 2014; 29: 185–194. doi: 10.3803/EnM.2014.29.2.185 25031892 10.3803/EnM.2014.29.2.185 PMC4091492

[54] Guo ZX, Wang YF, Feng X, Bao C, He Q, Bao LL, et al Rapamycin inhibits expression of ELOVL 1 and synthesis of docosahexaenoic acid in bovine mammary epithelial cells. Asian-Australasian Journal of Animal Sciences. 2016. 10.5713/ajas.15.0660 PMC5088386 26954224

[55] Rai M, Katti P, Nongthomba U. Spatio-temporal coordination of cell cycle exit, fusion and differentiation of adult muscle precursors by Drosophila Erect wing (Ewg). Mech Dev. 2016 doi: 10.1016/j.mod.2016.03.004 27039019 10.1016/j.mod.2016.03.004

[56] Zhang RP, Liu HH, Liu JY, Hu JW, Yan XP, Wang DM, Li L, Wang JW. Transcriptional profiling identifies location-specific and breed-specific differentially expressed genes in embryonic myogenesis in Anas platyrhynchos. PLoS One. 2015 12 2;10(12):e0143378 doi: 10.1371/journal.pone.0143378 eCollection 2015. 26630129 10.1371/journal.pone.0143378 PMC4667915

