# Supplementary material for: Deep RNA sequencing of pectoralis muscle transcriptomes during late-term embryonic to neonatal development in indigenous Chinese duck breeds
Source: PLoS One. 2017 Aug 3;12(8):e0180403. doi: 10.1371/journal.pone.0180403 (PMC5542427; doi:10.1371/journal.pone.0180403)
Supplement: S1 Fig — (DOCX) [file pone.0180403.s009.docx]

**Summary:** The two breeds presented consistent increases in body weight from E17, the body weights of the Gaoyou ducks at E17, E21, and E25 were significantly higher than those of the Jinding ducks. Significant effects of breed and age and their interaction were observed for the body weight of ducks during early development. The PM mass for the two breeds continued to increase at E17, but it did not increase from E21 to E27. The PM mass of Jinding ducks at 7 days even similar to the level detected at E21. The PM masses of Gaoyou ducks were significantly higher than those of Jinding ducks. Significant breed and age effects and interactions were observed for PM mass during early development. There was no sex effect were observed in PM.

Fig 1 Profiles of body weight (left), PM weight (right) during early development in ducks.

Notes: PM represents pectoralis muscle, n = 12–16.
